# ESCRT-dependent control of membrane remodelling during cell division

**DOI:** 10.1016/j.semcdb.2017.08.035

**Published:** 2018-02

**Authors:** Caroline Louise Stoten, Jeremy Graham Carlton

**Affiliations:** Division of Cancer Studies, King’s College London, London SE1 1UL, UK

**Keywords:** ESCRT, Endosomal sorting complex required for transport, Cytokinesis, Abscission, Nuclear envelope

## Abstract

The Endosomal Sorting Complex Required for Transport (ESCRT) proteins form an evolutionarily conserved membrane remodelling machinery. Identified originally for their role in cargo sorting and remodelling of endosomal membranes during yeast vacuolar sorting, an extensive body of work now implicates a sub-complex of this machinery (ESCRT-III), as a transplantable membrane fission machinery that is dispatched to various cellular locations to achieve a topologically unique membrane separation. Surprisingly, several ESCRT-III-regulated processes occur during cell division, when cells undergo a dramatic and co-ordinated remodelling of their membranes to allow the physical processes of division to occur. The ESCRT machinery functions in regeneration of the nuclear envelope during open mitosis and in the abscission phase of cytokinesis, where daughter cells are separated from each other in the last act of division. Roles for the ESCRT machinery in cell division are conserved as far back as Archaea, suggesting that the ancestral role of these proteins was as a membrane remodelling machinery that facilitated division and that was co-opted throughout evolution to perform a variety of other cell biological functions. Here, we will explore the function and regulation of the ESCRT machinery in cell division.

## 1 Introduction

**Fig. 1:**
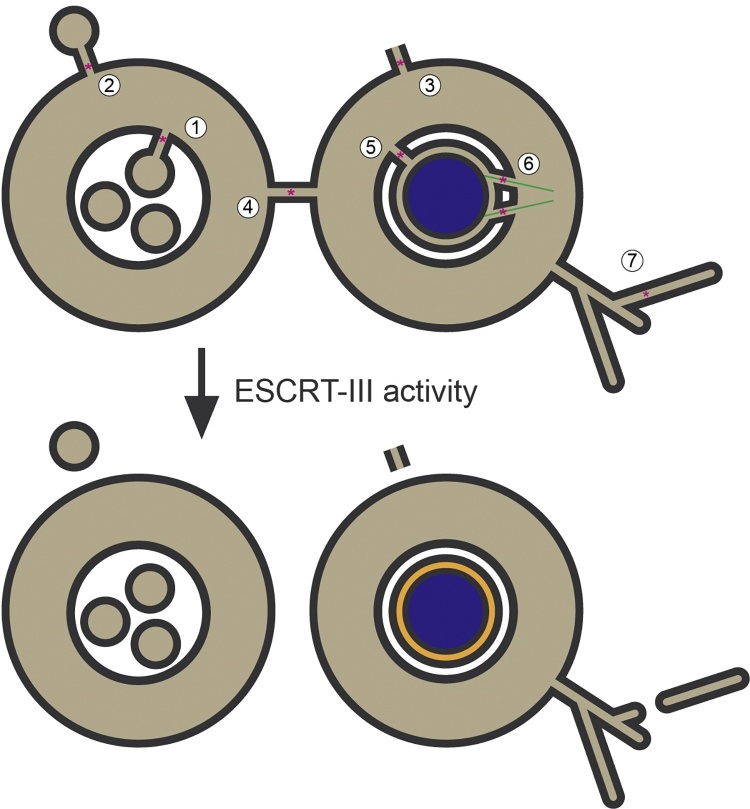
Topological equivalence of membrane ESCRT-dependent membrane remodelling events. ESCRT-III is a membrane fission machinery that regulates a series of topologically equivalent membrane remodelling events, namely biogenesis of intraluminal vesicles on a class of late endosomes called multivesicular bodies (1), release of enveloped retroviruses such as HIV-1 (2), repair of damaged plasma membrane (3), abscission during cytokinesis (4), sealing of holes in the nuclear envelope (5), depolymerisation of spindle microtubules (6) and neuronal pruning (7). In addition, ESCRT-III has also been described to regulate NPC quality in *S. cerevisiae* (not depicted; see Fig. 5). Sites of ESCRT-III activity indicated by magenta asterisks. Sealing of nuclear envelope allows nuclear compartmentalisation, indicated in orange.

The Endosomal Sorting Complex Required for Transport (ESCRT) proteins form a series of multi-subunit protein complexes that assemble into a cargo sorting and membrane remodelling machinery [1], [2]. Whilst initially discovered as regulators of cargo sorting on endosomes [3], [4], [5], it is becoming apparent that these complexes play essential and, until recently, relatively unappreciated roles in cell division [6], [7], [8]. The ESCRT machinery also plays essential roles in release of enveloped retroviruses and extracellular vesicles, in pruning neuronal processes, repairing both plasma and nuclear membranes and in regulating the quality of nuclear pore components (Fig. 1) [9], [10], [11]. Despite this apparent breadth of functions, the processes controlled by the ESCRT machinery share a requirement for a specific membrane remodelling event for their completion; the separation of membranes that were previously connected by a cytoplasm-filled channel (Fig. 1). We will not discuss here these myriad cellular processes and refer readers to a series of excellent reviews in this volume. Rather, we will focus here on emerging roles for the ESCRT machinery in cell division, with emphasis on ESCRT-III-dependent mitotic membrane remodelling.

## 2 Membrane and cytoskeletal remodelling events during division

**Fig. 2:**
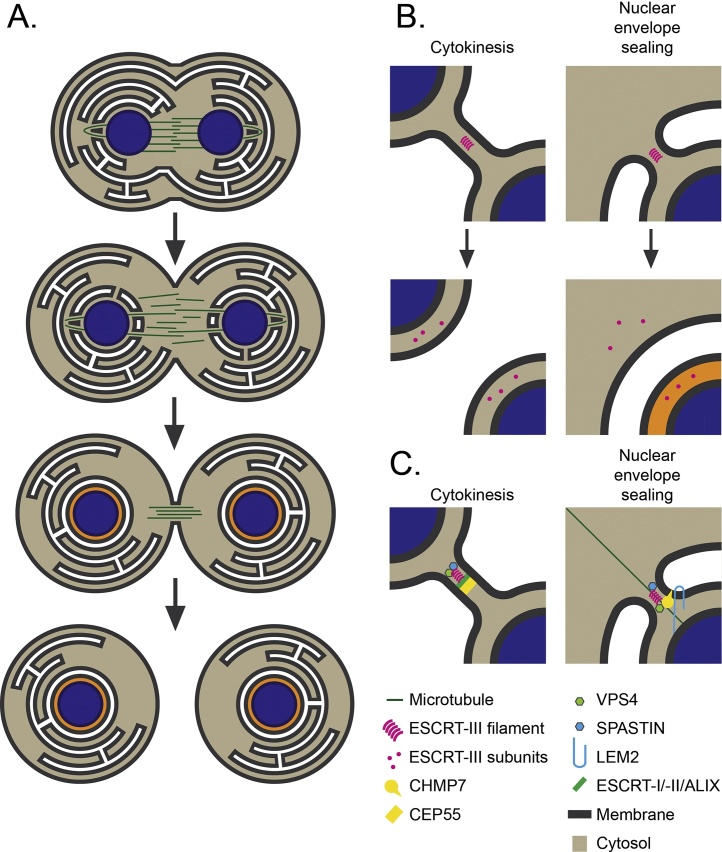
Roles for ESCRT-III in membrane remodelling during division. A. Membrane remodelling events during mitosis. As cells complete division, daughter nuclei are enclosed by sheets of ER to generate the nuclear envelope. Where these sheets meet in three dimensions, and where these sheets negotiate spindle microtubules, they leave small holes that are sealed by ESCRT-III dependent annular fusion. Completion of annular fusion permits nuclear compartmentalisation (indicated by nucleoplasm depicted in orange). Nuclear envelope regeneration happens concurrently with ingression of a cleavage furrow between daughter nuclei, leaving cells connected by a thin tube of membrane and compressed spindle microtubules, called the midbody. The midbody is severed through ESCRT-III-dependent abscission. B. Toplogical equivalence and consequences of ESCRT-III function during ESCRT-III-dependent membrane remodelling during mitotic events. C. Molecular interactions that govern ESCRT-III assembly at the midbody and at the reforming nuclear envelope. CEP55 recruits ESCRT-III to the midbody via either ALIX or ESCRT-I/ESCRT-II. ESCRT-III coordination of the microtubule severing enzyme spastin permits disassembly of midbody microtubules. CHMP7 initiates assembly of ESCRT-III at sites of annular fusion through engagement of ER-membranes and the chromatin-binding INM protein LEM2. ESCRT-III coordination of the microtubule severing enzyme spastin permits disassembly of spindle microtubules.

To allow cell division to proceed, cells must undergo a dramatic and complex restructuring of their membranes and of their cytoskeleton (Fig. 2A). The examples given below relate largely to metazoan mitosis, although similar physical rearrangements occur during meiosis. During cell division, cells must dismantle and then reassemble their microtubule cytoskeleton to form a spindle that allows motor-driven segregation of their duplicated genome. Rearrangements of the microtubule cytoskeleton occur at mitotic onset in parallel with the separation and migration of centrosomes, which were duplicated alongside chromatids in S-phase, to opposing sides of the nucleus [12]. Mammalian cells undergo an open mitosis and mitotic commitment results in phosphorylation and disassembly of lamins, inner nuclear membrane proteins and chromatin associated factors such as Barrier-to-Autointegration Factor (BAF) and Histone H3. This breakdown allows disassembly of connections between the nuclear lamina and the nuclear envelope, a bi-layered organelle that is continuous with the endoplasmic reticulum (ER) [13], [14]. Nuclear envelope disassembly is assisted by alterations in phospholipid synthesis that favour remodelling of this organelle and forces exerted by the cytoskeleton that enable its breakdown [13]. Nuclear envelope membrane proteins, including nuclear pore components that were similarly disassembled through mitotic phosphorylation, are released into the mitotic ER, and differential identity is lost between these organelles [13], [14]. Growing spindle microtubules, now being assembled from microtubule polymerisation at opposing centrosomes, latch onto mitotic kinetochores with amphitelic attachments ensuring bi-orientation at the metaphase plate [12]. The plus-ends of spindle microtubules that don’t engage kinetochores interdigitate at the centre of the cell, forming the characteristic diamond-shaped spindle. Satisfaction of the spindle assembly checkpoint (SAC) permits motor driven segregation of sister chromatids [12] and marks the onset of mitotic exit. During late anaphase and progress into telophase, an acto-myosin rich cleavage furrow ingresses between forming daughter cells, constricting the plasma membrane and, together with the action of bundling proteins such as Protein Regulator of Cytokinesis-1, condense the spindle into a tight bundle of microtubules that will form a structure called a midbody [15]. Concomitantly, sheets of mitotic ER are deposited upon the chromatin through interaction of chromatin binding proteins such as Heterochromatin Protein-1 and Barrier to Autointergration Factor (BAF), with the Lamin-B receptor and LEM-domain containing family of inner nuclear membrane (INM) proteins including MAN1 and Lamina-associated polypeptide 2β (LAP2β). Interaction of the nucleoporins ELYS/MEL-28, NDC1 and POM121 with chromatin can also assist ER-deposition [16], [17]. ER-sheets enveloping the now-decondensing chromatin are sealed through the process of annular fusion to allow regeneration of the nucleus through Nuclear Pore Complex (NPC)-driven re-compartmentalisation and subsequent re-establishment of separate nuclear envelope and ER identities [18], [19] (Fig. 2A and B). Alongside reconstruction of the nuclear envelope, acto-myosin-driven ingression of the cleavage furrow and formation of the midbody, the chromatin fully decondenses and the cell prepares for separation of its cytoplasm through the process of cytokinesis. During this process, the tubulin-rich midbodies are progressively thinned through removal of microtubules, leaving daughter cells tethered by a thin tube of membrane. This membrane is severed through the process of cytokinesis, resulting in two functional daughter cells [8], [20] (Fig. 2A and B). Importantly, the topology of membrane fission required for sealing holes in the nuclear envelope (annular fusion) and cytokinesis are identical (Fig. 2B).

Whilst the above description describes the situation in metazoans, related remodelling events occur in all organisms. However, key players and necessary physical processes can differ markedly. For example, yeast such as *S. cerevisiae* or *S. pombe* undergo a closed mitosis, assembling an intra-nuclear spindle without breaking open their nuclear envelopes [21]. *S. japonicus*, a fission yeast strain related to *S. pombe* undergoes a semi-open mitosis and provides an important comparative evolutionary model for analysis of mitotic programmes [22], [23]. Whilst we will discuss below the role of the ESCRT machinery in metazoan mitotic events, it is important to note that many mitotic phenotypes described have not been observed in yeast, and this may reflect the engagement of distinct mitotic programmes in these organisms and the circumvention of the ESCRT machinery by fungal-specific processes such as synthesis of a chitin-rich cell wall that participates in cleavage furrow ingression and cytokinesis. Roles for the ESCRT machinery (specifically the ESCRT-III complex) in division have, however, been observed in *Archaea* [24], [25], organisms separated from humans by over 2.5 billion years of evolution and devoid of internal membranes. These findings suggest that the ancestral role for the ESCRT machinery is in cell division, and that these proteins have been co-opted throughout evolution to perform a series of topologically related membrane remodelling events that allowed the development of multicellular organisms, and facilitated cellular exploitation by pathogens.

## 3 The ESCRT machinery – a subdivision of labour

Whilst not the goal of this review, it is perhaps helpful to introduce the individual complexes and subdivision of activities within the ESCRT machinery. In the context of endosomal sorting, and learning from examples described in yeast, the ESCRT machinery performs both cargo sorting and membrane remodelling functions. There exist 4 ESCRT complexes (Table 1; ESCRT-0, ESCRT-I, ESCRT-II and ESCRT-III) and a variety of accessory proteins that assist ESCRT function. ESCRT-I is a rod-shaped heterotetrameric complex [26] that interacts with a variety of adaptor proteins to localise this machinery to subcellular localisations [27], [28]. ESCRT-II is a Y-shaped hetrotetramer formed from a 1:2:1 complex of Vps22/EAP20, Vps25/EAP30 and Vps36/EAP45 that links ESCRT-I to ESCRT-III [29], [30], [31], [32], [33]. ESCRT-I and ESCRT-II contain ubiquitin-binding domains [34], [35], [36], [37] that participate in the transfer of ubiquitinated cargo onto nascent intraluminal vesicles. These complexes act at the bud neck of invaginating vesicles [38] and are localised to endosomal membranes through interaction with a 4th ubiquitinated-cargo binding complex, post-hoc christened ESCRT-0 [1], [2]. ESCRT-0 is a complex of Vps27 and Hse1 (and of Hepatocyte Growth Factor Regulated Tyrosine Kinase Substrate (HRS) and Signal Transducing Adaptor Molecule (STAM)-1 and 2 in mammalian cells). A PtdIns(3)P-binding FYVE (Fab1, YOTB, Vac1, EEA1) domain in Vps27/HRS localises ESCRT-0, and thus the entire machinery, to PtdIns(3)P-rich endosomes [2], [39], [40]. Vps27/HRS and Hse1/STAM both contain ubiqitin-binding Vps27, HRS and STAM (VHS) domains and additional uniquitin-interacting motifs (UIMs), highlighting ESCRT-0 as a critical endosomal ubiquitin cargo receptor [41]. In yeast, an interaction between Vps28 in ESCRT-I and an insertion in the ubiquitin-binding GLUE (GRAM-like Ubiquitin-binding in EAP45) domain of Vps36 forms a PtdIns(3)P binding split-pH domain that recruits this complex to membranes [32]. The situation in mammalian cells is a little less clear, but a 3-phosphoinositide-binding GLUE domain within VPS36 may play a similar role [36]. ESCRT-II can bind directly to the Vps20/CHMP6 subunit of ESCRT-III, and so links upstream ESCRT activity with the proposed membrane remodelling complex. It should be noted, however, that whilst ESCRT-II plays essential functions in Multivesicular Body (MVB) sorting in yeast [4], [42], roles for ESCRT-II in mammalian cells have been a little confusing, with links between ESCRT-I and ESCRT-III suggested to be provided by adaptor proteins such as ALG-2-Interacting Protein X (ALIX) [43], [44], [45], [46] and Histidine-Domain Protein Tyrosine Phosphatase (HD-PTP) [47], [48], [49], both of whom bind directly to the ESCRT-I component Tumour Susceptibility Gene-101 (TSG101) and filament-forming subunits of ESCRT-III [50]. ESCRT-II has been found dispensable for Human Immunodeficiency Virus-1 (HIV-1) release and endosomal sorting [51], [52]. However, recent data suggest an alternative explanation in that ESCRT-II behaves as an assistive linker between ESCRT-I and −III during cytokinesis [53], [54] and viral release [55], [56], suggesting that cells have evolved parallel mechanisms to recruit ESCRT-III. ESCRT-III itself comprises a polymer of small self-assembling proteins, which in mammalian cells are known as CHMPs (Charged Multivesicular Body Proteins/Chromatin Remodelling Proteins) and the CHMP-like protein, Increased Sodium Tolerance-1 (IST1) [2], [5], [57], [58]. CHMPs contain 5, or sometimes 6, recognisable alpha helices and exist in soluble or filament forming conformations. Transition between states is regulated by displacement of a C-terminal regulatory helix which is thought to drive assembly and membrane remodelling [42], [59], [60], [61]. Notably, roles for ESCRT-III proteins in *Archaea* division was described in strains lacking FtsZ or Mre1, the prototypical actin and tubulin paralogues, suggesting that ESCRT-III may represent an ancestral filament forming machinery that may function in place of these cytoskeletal members [24], [62].

**Table 1:** Gene names of ESCRTs from the organisms discussed in this review. Aliases, typically used in mammalian systems, are provided. Fly and worm orthologues compiled from Flybase beta (FB2017_93) and Wormbase WS259. Where orthologues were predicted, but not annotated, sequence identifiers were used. *Usp-50* and *Ego-2*’s equivalence to *UBPY* and *PTPN23* was from [180].

	*S. cerevisiae*	*H. sapiens*	*C. elegans*	*D. melanogaster*	*Aliases*
ESCRT-0	*Vps27*	*HGS*	*hgrs-1*	Hrs	*HRS*
	*Hse1*	*STAM1/STAM2*	stam-1	Stam	

ESCRT-I	*Vps23*	*TSG101*	*tsg-101*	Tsg101	
	*Vps28*	*VPS28*	*vps-28*	Vps28	
	*Vps37*	*VPS37A/VPS37B/VPS37C/VPS37D*	*vps-37*	Vsp37a/Vps37b	
	*Mvb12*	*UBAP/MVB12A/MVB12B*	*mvb-12*	Mvb12	

ESCRT-II	*Vps25*	*VPS25*	*vps-25*	Vps25	*EAP20*
	*Vps22*	*VPS22*	*vps-22*	Vps22/Larsen (Lsn)	*EAP30*
	*Vps36*	*VPS36*	*vps-36*	Vps36	*EAP45*

ESCRT-III	*Did2/Vps46*	*CHMP1A/CHMP1B*	*did-2*	Chmp1	
	*Vps2*	*CHMP2A/CHMP2B*	*vps-2*	Chmp2b	
	*Vps24*	*CHMP3*	*vps-24*	Vps24	
	*Snf7*	*CHMP4A/CHMP4B/CHMP4C*	*vps-32*	shrub	
	*Vps60*	*CHMP5*	*vps-60*	Vps60	
	*Vps20*	*CHMP6*	*vps-20*	Vps20	
	*Chm7*	*CHMP7*	T24B8.2	CG5498	
	*Ist1*	*IST1*	*istr-1*	CG10103	

ESCRT	*Bro1*	*PDCD6IP/PTPN23*	*alx-1/ego-2*	ALiX	*ALIX/HD-PTP*
Associated	*Doa4*	*UBPY/STAMBP*	*usp-50*	Usp8/CG2224	*UBPY/AMSH*
	*Vta1*	*VTA1*	T23G11.7	Vta1	*LIP5*
	*Vps4*	*VPS4A/VPS4B*	*vps-4*	Vps4	*SKD1 (VPS4B)*
		*SPAST*	*spas-1*	Spastin	*Spastin*
		*CC2D1A/CC2D1B*	Y37H9A.3	Lethal (2) Giant Discs (Lgd)	
		*MITD1*	Y66D12A.10	CG14985	

CHMPs polymerise into spiral filaments on membranes [63], [64], [65], [66] and it is thought that these spirals assemble within the membranous stalks described in Fig. 1. ESCRT-III activity is predicted to result in severing of these stalks and separation of the previously joined membranes, permitting vesicle, viral, cell, or nuclear membrane separation [1], [6]. Notably, ESCRT-III proteins contain C-terminal peptide stretches called MIT-domain Interaction Motifs (MIMs) that engage the Microtubule Interaction and Trafficking (MIT)-domain of an evolutionarily conserved AAA-ATPase called VPS4 [67], [68], [69]. VPS4 represents the sole energy consuming element of this pathway and is necessary for recycling and disassembling ESCRT-III filaments. The exact mechanism of ESCRT-III dependent membrane separation remains a matter of intense debate; in recent years the discovery that ESCRT-III spirals force individual CHMP subunits to adopt energetically unfavourable conformations has allowed the proposal that these spiral filaments form an elastic spring that can store energy and later release it for membrane deformation, or indeed, scission [65], [66], [70]. Likewise, ATP consumption by VPS4 has been suggested to both disassemble [71] and remodel [72] the ESCRT-III filament, which may contribute toward its activity in membrane fission. A variety of suggestions for VPS4-dependent regulation of ESCRT-III have been proposed, culminating in biophysical models such as ‘dome [73]’, ‘whorl [33]’ or ‘reverse dome [1]’ whereby progressive constriction of an ESCRT-III filament, capped by a dome-shaped subcomplex of CHMP2/CHMP3 is thought to lower the energetic barrier of severing the membranous stalk and achieving membrane fission [73]. Alternate models, such as the buckling model proposed by Hurley [1], attempt to reconcile observations that are inconsistent with the dome models (namely that in a variety of systems, ESCRT-III filament growth is outward, rather than constrictive [66]) and attempts to integrate hypotheses concerning the elastic nature of these filaments. In these models, radial growth of the filament allows the accumulation of energy at unfavourable curvatures which, upon breaching some elastic limit, is released to ‘buckle’ and sever the membrane.

As well as core ESCRT-III subunits, evidence is emerging that polymerisation of ESCRT-III may be controlled through the engagement of regulatory Coiled-Coil and C2-domain containing 1A/1B proteins (CC2D1A/CC2D1B; Lethal Giant Discs, (Lgd) in *D. melanogaster*). These proteins bind specifically to the Shrub/CHMP4 N-terminus proteins through their third DM14 domain [74], [75]. In mammalian cells, this interaction acts to suppress ESCRT-III dependent function, for example, during HIV-1 release [74], [76], whereas in flys, Lgd has been suggested as a positive regulator of ESCRT-III function during endosomal sorting [77] and abscission [78]. Interestingly, crystal structures of the *D. melanogaster* CHMP4 orthologue (Shrub) showed that these proteins polymerise in a head-to-head orientation, with salt bridges and complementary inter-subunit electrostatic interactions driving filament assembly [79] (although in yeast, analogous inter-subunit interactions were supported by hydrophobic, rather than electrostatic contacts [80]). Interestingly, the polymerisation surface in Shrub is also the Lgd-binding surface, and the crystal structure of a Lgd-DM14-3/Shrub core fusion demonstrated that Shrub occluded the polymerisation surface, suggesting that these proteins act as allosteric inhibitors of ESCRT-III polymerisation [75]. A similar situation is thought to occur with mammalian proteins, whereby CC2D1A suppresses CHMP4 polymerisation through analogous interactions [74]. CC2D1A/CC2D1B orthologues are absent from yeast, suggesting that acquisition of this electrostatic polymerisation interface, and the ability to control filament assembly through these regulatory proteins, represents an evolutionary adaptation of this pathway as cells gained complexity. It should be noted that if these proteins do act as negative regulators of ESCRT-III polymerisation, then their absence may lead to inappropriate ESCRT-III polymerisation, which may explain the conflicting reports as to whether these proteins positively or negatively regulate ESCRT-III function.

## 4 Adaptors for localising ESCRT-III

The exact mechanism by which membranous stalks are severed and membranes separated awaits experimental validation, however, it is clear that ESCRT-III is the main driver of this process. In a cell biological context, this machinery is recruited to distinct sites of action through interaction with a variety of adaptor proteins [6], [11]. In the context of viral release, Late-domains (L-domains) within various viral structural proteins have evolved to bind directly to ESCRT-I and accessory proteins. For example, the primary L-domain of HIV-1, a PTAP motif within Gag-P6, mimics the TSG101-binding motif (PSAP) within HRS and directly binds TSG101′s UEV domain. HIV-1 also contains an auxiliary L-domain, an LYPxL motif within Gag-P6, that binds the ESCRT-III adaptor protein ALIX [2], [81]. In this way, enveloped retroviruses recruit the ESCRT-III machinery to budding sites to facilitate their cellular egress and the evolution of auxiliary L-domains ensures robust recruitment of this machinery. For membrane repair, ALG2 is necessary for recruitment of this machinery to the plasma membrane [82], [83]. In the context of cytokinesis, a specialised midbody protein, Centrosomal Protein of 55 kDa (CEP55), makes direct interactions with TSG101 and ALIX to facilitate recruitment of ESCRT-III to this structure (Fig. 2C) [45], [46], [84], [85]. The ESCRT machinery thus represents a transplantable membrane remodelling complex that is targeted to a variety of subcellular localisations through interaction with distinct adaptor proteins to enable an ESCRT-III-catalysed membrane separation.

## 5 ESCRT-dependent regulation of cytokinetic abscission

In the final stages of cytokinesis, the midbody (a membrane and microtubule-rich intercellular bridge connecting daughter cells) is severed through the process of abscission. This process bears a topological equivalence to the membrane separation events performed by ESCRT-III (Figs. 1 and 2B). A centralspindlin-interacting protein, CEP55, plays an essential role in coordinating a machinery able to perform cytokinetic abscission [86], [87]. The midbody contains a number of geographical zones, well described by [88], and we refer to the midbody as the entire intercellular bridge and the central ‘dark zone’ as the Flemming body. CEP55 localises to centrosomes in interphase and during early mitosis [86], [87], [89] and later transitions to the Flemming body during mitotic exit. Whilst data initially suggested that Polo-like Kinase-1 (PLK1) was a positive regulator of this CEP55 dependent recruitment [86], [89], more recent data indicates that PLK1 phosphorylation of CEP55 prevents it from both interacting with the central spindle and being prematurely recruited to the midbody. CEP55 translocation to the Flemming body is permitted as PLK1 is degraded during mitotic exit [90]. CEP55 directly binds two ESCRT components through interaction with conserved GPP motifs in both ALIX and TSG101 [45], [46], [84]. Intriguingly, ALIX and TSG101 compete for the same site in the hinge region of CEP55 [85], suggesting that, just as many viruses employ primary and auxiliary L-domains [28], robust mechanisms exist to ensure proper recruitment of the ESCRT machinery by CEP55. ALIX’s BroI domain makes direct contact with the C-termini of the CHMP4 subunits of ESCRT-III and, in HeLa cells, this axis is essential for proper completion of cell division [45], [46], [50]. ALIX itself is subject to phospho-regulation, with mitotic phosphorylation of residues in ALIX’s C-terminal Proline-Rich Region causing a conformational change that triggers interaction with CHMP4 proteins and cytokinetic abscission [91]. These data suggest that phosphorylation of ESCRTs can be a trigger for coordinating recruitment and activity of these complexes during mitosis.

Whilst ALIX seems the major adaptor for cytokinesis in HeLa cells, the existence of ESCRT-II dependent cytokinesis routes that are exposed in the absence of an ALIX:ESCRT-III interaction highlight the robustness of this axis for the completion of cell division [53]. In both cases, direct recruitment by CEP55:ALIX:CHMP4 or indirect recruitment through a TSG101:ESCRT-II:CHMP6 route, the placement of ESCRT-III at the midbody is thought to catalyse the final membrane separation [92], [93]. In addition to the core members, whilst dispensable for MVB sorting and HIV-1 release, mammalian IST1 plays an essential role in abscission [94], [95], indicating that process-specific ESCRT-III complexes are assembled. Additional ESCRT-III requirements specific for abscission have been exposed through the discovery that the MIT-domain containing protein, Microtubule Interacting and Trafficking Domain Containing 1 (MITD1), interacts with ESCRT-III through binding CHMP1A, CHMP1B, CHMP2A and IST1, and is necessary for abscission [96], [97]. MITD1 contains a membrane-binding Phospholipase-d-like fold and the ability of MITD1 to bind membranes through this domain was essential for abscission [96], suggesting that this protein may function as an ESCRT-III-membrane tether during abscission. In the case of cytokinetic abscission, the membrane remodelling activity of ESCRT-III is necessarily coordinated with localised disassembly of microtubules in the midbody. To effect this, the MIT-domain containing AAA-ATPase, Spastin, is recruited to the midbody through coordination of MIMs in ESCRT-III subunits such as CHMP1B [98], [99] and provides an activity essential for microtubule clearance and cytokinetic abscission. In this manner, ESCRT-III coordinates membrane and cytoskeletal remodelling events essential for the completion of cell division (Fig. 2C).

## 6 Dynamic nature of ESCRT-III at the abscission site

Excitingly, using state of the art electron tomography, spiral filaments have been visualised at the abscission site that may correspond to filaments of ESCRT-III [92]. Whilst the existence of these filaments was ESCRT-III [92] and VPS4 [72] dependent, their 17 nm diameter is at odds with observed sizes for homomeric ESCRT-III filaments. However, it is possible that these thicker filaments represent ESCRT-III *in-toto* – a situation that has not yet been achievable *in-vitro*. Indeed, co-polymers of CHMP1B and the N-terminal domain of IST1 have recently been described, that result in 24 nm diameter filaments [100]. However, definitive proof that these cytokinetic filaments are indeed ESCRT-III spirals awaits experimental validation. Building on these results, and the recent demonstration from the Roux lab that ESCRT-III filaments behave like elastic springs, using elastic potential energy from filament assembly to remodel the membrane [66], Gerlich, Roux and colleagues [72] have recently discovered that cytokinetic ESCRT-III filaments are more dynamic than previously thought. Using high-resolution microscopy and photobleaching approaches, they discovered that subunit exchange occurs between cytoplasmic and membrane-bound, midbody-assembled pools of CHMPs, with filament makeup turning over almost completely during residency at the midbody. These data indicate that the ESCRT-III filament is actively remodelled as cells progress through cytokinesis. Recruitment kinetics for ESCRT-III members (CHMP2B, CHMP3 and CHMP4B) were indistinguishable, with these subunits being recruited gradually over the 40 min prior to abscission, suggesting that co-polymeric filaments assemble during cytokinesis. Dynamic subunit exchange was dependent upon VPS4′s ATPase activity and VPS4 itself was necessary for midbody constriction, suggesting that dynamic subunit remodelling creates the force necessary to bring the membranes together for abscission [72].

Filament remodelling and subunit exchange could also be observed in-vitro, using purified yeast proteins, and here, the authors observed that Vps24 and Vps2 could suppress Snf7 polymerisation, suggesting that these subunits act to regulate assembly of the membrane remodelling filament. However, rather than acting as a ‘cap’ to the growing Snf7 spiral, as has been previously proposed [59], these proteins polymerised alongside Snf7 spirals and appeared to bundle the Snf7 filaments into a disc like morphology that suppressed further Snf7 growth [72]. Again, Vps4 remodelled these filaments to allow further Snf7 polymerisation. Thus, one exciting new role for Vps4 that this study exposes is its function as a quality control mechanism, disassembling non-productive filament assembly and removing the brake that Vps2/Vps24 filaments seem to impose on Snf7 filament growth. These data have profound implications for our understanding of how the ESCRT-III filament behaves, both during cytokinesis and in all other ESCRT-III-dependent processes. Moreover, these data suggest that VPS4 plays an active role in constriction and filament remodelling rather than the disassembly role most often ascribed to it. If it is possible to remove and replace subunits from within a pre-existing filament, what effect does this have on the rigidity or force imposing properties of these filaments? Does subunit exchange allow for filament remodelling to better match the geometric constraints of the membrane environment being remodelled?

## 7 Maturation of the midbody

**Fig. 3:**
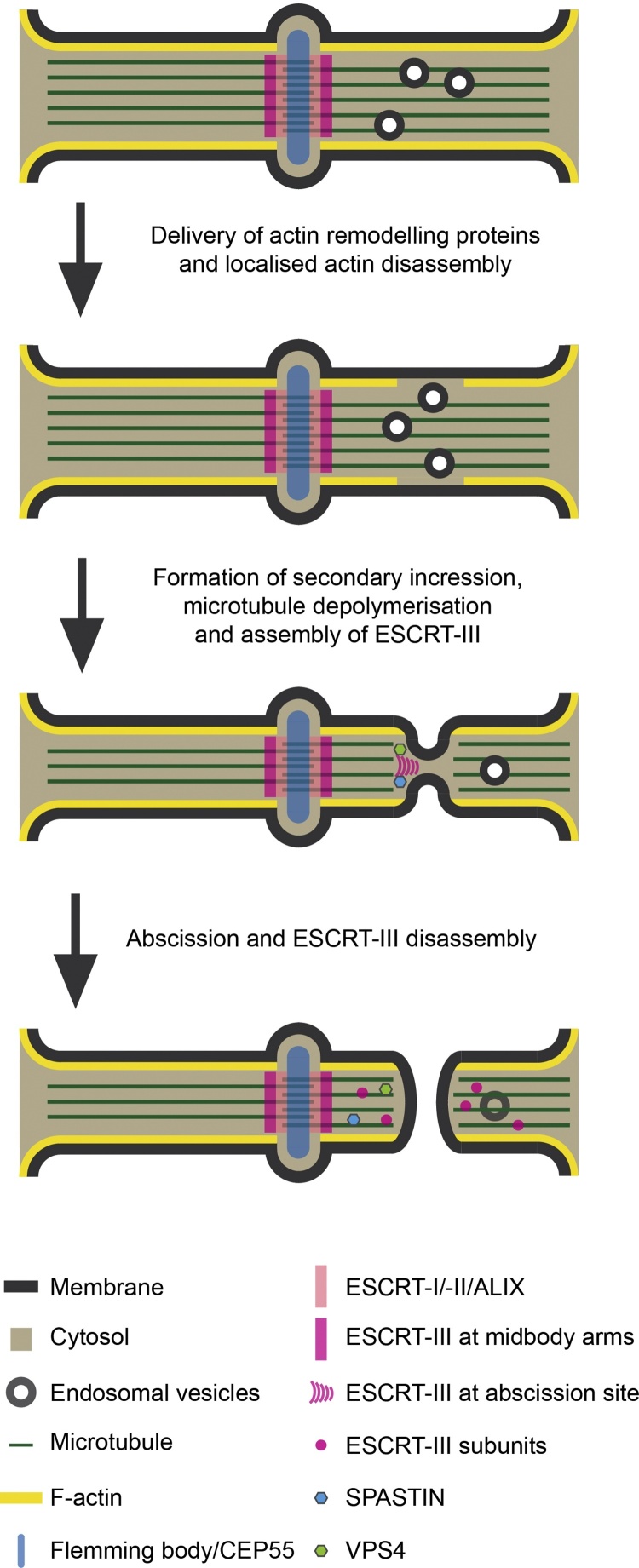
Morphological changes during midbody maturation. The midbody tethers daughter cells at the end of division, and upstream ESCRT-I and ALIX components assemble at the Flemming body. Prior to abscission, ESCRT-III components assemble in two rings either side of the Flemming body. Rab11-FIP3 positive vesicles and delivery of the actin remodelling proteins p50RhoGAP, SCAMP2/3, OCRL, and MICAL1 are necessary to remodel the actin cytoskeleton and form a secondary ingression that narrows the midbody to permit subsequent ESCRT-III assembly at the site of abscission. Again, ESCRT-III coordination of Spastin assists in disassembling the midbody microtubules, thus coordinating membrane and cytoskeletal remodelling.

During cytokinesis, progression from midbody formation to abscission takes upwards of an hour (in cultured HeLa cells), rendering abscission a G1 event [101]. During this time, midbodies become thinner and undergo a series of poorly understood morphological changes deemed ‘maturation’, until the signal to complete cytokinesis is triggered. In some cell types, for example, Germline Stem Cells (GSCs), abscission can be delayed for many hours and occurs well after mitosis completes [102], [103]. Just as live cell imaging revealed the transient appearance of ESCRT-III members immediately prior to virion release [104], [105], the peak of ESCRT-III components at the midbody occurs just prior to cell separation [72], [92], [93], [106]. Interestingly, the initial localisation of ESCRT-III subunits appears to be to two rings on each side of the Flemming body, but this does not represent the actual cut site. The site of abscission is specified approximately 1 μm along the midbody arms at a thinning, or secondary ingression, where the midbody diameter is reduced from circa 1 μm to 0.1–0.2 μm, which may be a more geometrically appropriate structure for ESCRT-III to act on (Fig. 3). How ESCRT-III transitions from the initial assembly site to the cut site remains unclear – whether a spiral filament extends this distance, or whether a discrete secondary filament is formed awaits experimental validation [93].

Progressive removal of tubulin filaments by microtubule-severing ATPases such as Spastin [98], [99], is likely to facilitate midbody thinning, but is unlikely to account fully for the delay in abscission initiation as the secondary ingression and localised removal of tubulin for abscission occurs in a narrow zone within a still-tubulin-positive midbody. Whether two distinct tubulin remodelling events (thinning and local removal) are required for abscission remains unknown. In the case of migratory cells with room to spread, a lengthening of the midbody is also observed, and, as discussed below, this can influence the timing of abscission [107], [108].

As well as reorganisation of the microtubule cytoskeleton, the actin cytoskeleton must also be remodelled. F-actin plays a critical role in cleavage furrow ingression and has been observed to persist in the furrow until the time of abscission [109]. However, it has been unclear how actin is removed from the abscission site to enable abscission to proceed. Formation of the secondary ingression itself requires actin remodelling [110], suggesting that the actin cytoskeleton plays important roles in templating the sub-midbody structure to facilitate membrane abscission. Endosomal delivery of the actin remodelling protein p50RhoGAP and the Secretory Carrier-Associated Membrane Protein 2/3 (SCAMP2/3) transmembrane proteins, via Rab11 Family-Interacting Protein-3 (FIP3)-positive endosomes [110], are necessary to create the secondary ingression [110]. In addition, Rab35 [111], Occulo-Cerebro-Renal syndrome of Lowe (OCRL) [112] and Molecule Interacting with CasL-1 (MICAL1) [113] have been shown to participate in removal of cortical F-actin from the midbody, so facilitating abscission. MICAL1 is a monoxygenase that contributes to actin disassembly through direct oxidation of methionine residues on actin [114]. Echard and colleagues have recently discovered that F-actin accumulation in the midbody prevents CHMP4B-recruitment and that MICAL1 is recruited to, and activated at, midbodies through direct interaction with GTP-Rab35 [113]. In this manner, MICAL1 helps depolymerise F-actin, clearing the way for ESCRT-III assembly at the abscission site (Fig. 3).

Alongside changes to the actin and microtubule cytoskeletons, the plasma membrane also gets remodelled during cytokinesis. The plasma membrane lipid PtdIns(4,5)P_2_ plays an important role in cytokinesis [115], [116], [117] and cortical actin remodelling [118]. As well as participating in actin-removal, ORCL-dependent dephosphorylation of PtdIns(4,5)P_2_ [112], [119] is thought to contribute to cytokinesis progression, although whether its activity remains focussed at the midbody [112], or whether endosomal PtdIns(4,5)P_2_-dephosphorylation elevates the relative cytokinetic pool of this lipid [119] remains a matter of ongoing investigation. Other links between actin and plasma membrane remodelling include SCAMP3, which was necessary for actin remodelling, but can bind the ESCRT components HRS and TSG101 [120] and could thus play a role in stabilising these proteins at the midbody. Whilst HRS is most known for coordinating ESCRT-assembly on endosomes through its PtdIns(3)P-binding, FYVE domain, recent studies have also detected a midbody pool of PtdIns(3)P that recruited the FYVE-domain containing protein, FYVE-CENT to assist abscission [121]. In this light, reports of Class III PI 3-kinase dependent abscission [122] suggest that 3′-phosphorylated phosphoinositides may also contribute to midbody remodelling.

As such, an interplay between actin and microtubule cytoskeletons, and plasma membrane lipids, underlies the process of midbody maturation (Fig. 3). We believe that this remodelling acts as a molecular timer to delay engagement of the abscission machinery until the cell is ready to irreversibly split.

## 8 Checkpoint control of abscission timing

Mitosis follows a temporally coordinated unidirectional programme, with progression between sequential phases being rapid but with a series of control points inserted to ensure progression occurs only when subsequent stages have been performed satisfactorily. The major control point in mitosis, the SAC, delays anaphase onset until bi-orientation of sister chromatids is achieved. However, in recent years, an additional control point has been identified during the final stages of cytokinesis – the abscission checkpoint. This checkpoint is thought to allow the cell to resolve any post-SAC defects accompanying chromosome segregation, and appears to exploit the natural control mechanisms governing abscission timing and midbody maturation.

**Fig. 4:**
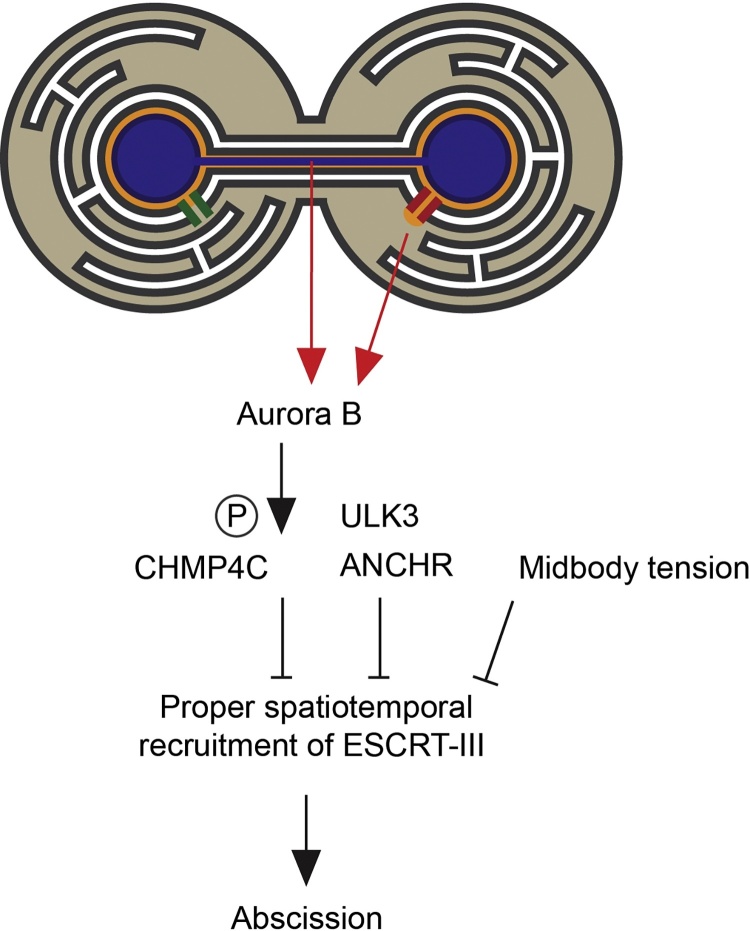
Role of ESCRT-III in the Aurora B mediated abscission checkpoint. Cartoon depicting triggers that initiate Aurora B-dependent abscission arrest. Elevated Aurora B downstream of chromosome segregation errors or NPC assembly defects (NPC in red, with indicated compartmentalisation breakdown, as per [179]) act to retard ESCRT-III-dependent abscission. Whilst a direct target of Aurora B is CHMP4C, ANCHR and ULK3 contribute to maintenance of this checkpoint. This checkpoint appears effected by spatiotemporal control over ESCRT-III assembly within the midbody; Aurora B phosphorylation directs CHMP4C to the Flemming body and ULK3 also drives ESCRT-III components onto the Flemming body. ANCHR acts to restrict VPS4 to the Flemming body and prevents it relocalising to the secondary ingression. Tension in the midbody can act to suppress ESCRT-III-assembly at the midbody and ESCRT-III-dependent abscission and this signalling acts though ULK3.

The existence of such a checkpoint was described first in yeast that exhibited an abscission delay in response to anaphase defects. This delay, deemed a control mechanism called NoCut, was shown to depend upon Aurora/Ipl1 in the spindle midzone and the presence of chromatin in the cleavage plane [123], [124]. Chromosome breakage was observed in the absence of NoCut, suggesting that it represents an adaptive response to chromosome segregation errors [123]. Similar checkpoint behaviour was observed in mammalian cells, where an Aurora B-mediated abscission checkpoint was activated in the presence of lagging chromosomes and acted to delay abscission to facilitate proper chromosome-segregation [109]. Sustained Aurora B activity, localised to the chromosome bridge, was revealed as the source of this abscission delay, suggesting the existence of Aurora B substrates that act to effect this checkpoint [109] (Fig. 4). Whilst the Aurora B-dependent cytokinesis checkpoint is activated in response to mitotic defects, this kinase also imposes temporal control in the context of normal cytokinesis [109]. This is particularly evident in GSCs, where cytokinesis is substantially delayed, leaving cells connected for extended periods by an inter-cellular bridge [103]. In *D. melanogaster* GSCs, ALIX and ESCRT-III are necessary for abscission [78], [125] and recruitment of CHMP4B/Shrub to the site of abscission occurs nearly 10 h after mitosis, suggesting the existence of regulatory networks that suppress untimely assembly of this machinery [102]. Aurora B appears part of this network and acts to prevent precocious abscission in these cells [102], likely by negatively regulating Shrub-dependent abscission [78]. Indeed, disruption of Aurora B advanced abscission in these cells in a manner that could be rescued by constitutively active Cyclin B [126]. In mammalian cells, Cyclin B2 localised to the midbody and positively regulated abscission [126], suggesting that these kinases may act together as a molecular timer regulating abscission timing.

In addition to Aurora B- and ESCRT-III-dependent control of abscission timing, it also appears that regulation of actin remodelling can contribute to this process. As described above, actin must be cleared from the midbody to allow cytokinesis to proceed [110], [113]. In cells in which the abscission checkpoint is engaged (for example, those bearing a lagging chromosome), actin persists in the furrow and accumulates at patches proximal to the midbody until the mis-segregating chromosome is resolved [109]. In GSCs, a secondary actin ring contributes to the abscission delay that maintains the intercellular bridge in these cells [102]. Although we know relatively little about how this regulation occurs, the actin cytoskeleton’s role in regulating midbody maturation and the progress to abscission appears integrated into the abscission checkpoint.

## 9 Molecular regulators of the abscission checkpoint

Whilst Aurora B may regulate abscission timing, the mechanistic basis for this is only recently coming to light. One Aurora B substrate has been revealed to be the ESCRT-III subunit, CHMP4C [106], [127]. CHMP4C is related to the major filament-forming CHMP4B subunit of ESCRT-III, although protein copy-number analysis suggests that it is expressed at 10-fold lower levels [128]. CHMP4C-depletion produces accelerated abscission, suggesting that, like Aurora B [102], [109], [126], this protein contributes to a regulatory delay in normal cytokinesis that allows proper midbody maturation [106]. CHMP4C is preferentially recruited to the midbody through the CEP55-ALIX route, rather than the CEP55:TSG101:ESCRT-II route [53], and contains a C-terminal insertion containing residues that can be phosphorylated by Aurora B, suggesting that it may be a CHMP4 paralogue that has evolved to accept Aurora B-mediated control. Whilst S210 represents the major phospho-acceptor [106], S214 and S215 also receive Aurora B-dependent phosphorylation [127]. Overexpression of CHMP4C (either WT [106], [127], phosphomimic, or phosphomutant forms [127]) impairs abscission. Cells reliant upon versions of CHMP4C unable to accept Aurora B phosphorylation display premature abscission, even in the presence of mis-segregating chromatin, suggesting that Aurora B-phosphorylated CHMP4C retards abscission [106] (Fig. 4).

The mechanism by which CHMP4C delays abscission is a matter of ongoing investigation; CHMP4C localises early in cytokinesis and occupies the Flemming body [106], whereas CHMP4B decorates the midbody arms [93], [106]. RNA-interference and rescue experiments employing phosphorylation-defective forms of CHMP4C suggest that Aurora B-mediated phosphorylation of CHMP4C prevents it migrating from the central region of the midbody to the midbody arms to trigger abscission [106]. Indeed, geographical segregation of abscission components within the midbody has been suggested as a mechanism through which abscission timing is regulated through other effectors too. A Flemming-body localised NoCut regulator called ANCHR (Abscission/NoCut Checkpoint Regulator) binds VPS4 through a MIM1 motif and retains VPS4 in the Flemming-body, preventing it from transitioning to the site of abscission [129]. In this way, the abscission checkpoint is effected through spatio-temporal control over ESCRT-III assembly and abscission (Fig. 4).

However, there are alternate explanations for the mechanistic basis of CHMP4C-dependent abscission control; the Chromosomal Passenger Complex component Borealin binds directly to CHMP4C’s N-terminus [127] and it has been proposed that this interaction impairs CHMP4C’s membrane remodelling activity, as evidenced by atomic force microscopy-visualised remodelling of bilayers in-vitro by CHMP4C [130]. These studies also revealed the presence of S210-phosphorylated CHMP4C in the spindle midzone and restricted to the Flemming body, whereas a S210/S214/S215 tri-phosphorylated signal decorated the midbody arms, suggesting a complex pattern of CHMP4C phosphorylation occurs during abscission progression and that phosphorylation may govern the geographical distribution of CHMP4C within the midbody [130]. Indeed, Aurora B-independent phosphorylation of CHMP4C has been reported during mitosis [108], and the identity of kinases involved in phosphorylation of, and functional roles for these phosphorylations on, this critical ESCRT-III subunit await identification.

The ESCRT machinery has evolved to include additional members that help regulate this checkpoint; the Unc51-like kinase3 (ULK3) can bind MIM-containing ESCRT-III components via tandem MIT-domains and phosphorylates these proteins [108]. Similarly to CHMP4C, ULK3 depletion accelerates normal abscission and blunts the response to abscission checkpoint activation. ULK3′s involvement in the abscission checkpoint was dependent upon its kinase activity and its ability to bind ESCRT-III components [108]. Similarly to the spatiotemporal control of ESCRT-III localisation described above, ULK3 overexpression drove retention of ESCRT-III components in the Flemming body, in a kinase-dependent manner and ESCRT-III subunits that couldn’t be phosphorylated by ULK3 failed to support abscission arrest in response to engagement of this checkpoint [108]. These data indicate that ULK3 is a critical component of the abscission checkpoint and that its kinase activity, along with that of Aurora B, is important for checkpoint activity.

## 10 Engagement of the abscission checkpoint

Thus the abscission checkpoint can be effected through regulation ESCRT-III − how then, is it engaged? Lagging chromosomes have been discussed as a mechanism for engaging an abscission delay, but there are a number of other events that are integrated into this checkpoint. Decatenation errors stemming from improper DNA-replication in S-phase can lead to ultrafine bridges (UFBs) of single-stranded DNA during anaphase [131]. UFBs fail to be detected beyond anaphase, suggesting that they may be resolved as mitosis progresses. Protein Kinase C Epsilon (PKCε) operates in metaphase to help resolve persistent catenation and suppress the formation of these UFBs [132]. A similar situation exists in yeast where Topoisomerase-2 or Condensin inactivation results in spindle stabilisation, engagement of the NoCut checkpoint in response to chromatin bridges and abscission delays to promote viability after replication stress. Strikingly, chromatin forced into the cleavage plane from artificial dicentric chromosomes did not activate NoCut, indicating that the nature of the bridged chromatin is key to engagement of this checkpoint [124], [133]. Unreplicated DNA lesions can be detected in cytokinetic nuclei and replication stress can also trigger abscission arrest through Ataxia telangiectasia and Rad3- (ATR) and Checkpoint Kinase 1- (CHK1) mediated engagement of the abscission checkpoint [134], indicating that these kinases participate in genome surveillance and mediate mitotic stress-induced abscission arrest. In mammalian cells, PKCε functions again in the response to chromatin in the cleavage plane, whereby it phosphorylates Aurora B to alter its substrate specificity, favouring phosphorylation of Borealin and engagement of the abscission checkpoint. Abscission control mediated through PKCε and Aurora B can again be bypassed through CHMP4C depletion, suggesting again that this ESCRT-III member is a key effector in this process [135].

A variety of stresses independent of DNA-replication or chromosome segregation can also engage the abscission checkpoint. Tension in the intracellular bridge delays abscission and tension release triggers ESCRT-III assembly, suggesting a biophysical mechanism exists to retard or accelerate abscission, depending on the physical environment sensed by the cells [107]. In the case where division results in daughter cells occupying spatially distinct areas, a delay in the final separation until cells are properly placed may assist organisation of tissue morphology [107]. A further stress that can impinge upon abscission dynamics is nuclear pore integrity. In recent years, remarkable control over abscission timing has been revealed through manipulations that impair proper functioning of the NPC [136]. Inhibiting NPC assembly, through expression of a truncated nucleoporin component, or depletion of NUP153 can trigger an Aurora B-dependent abscission arrest [137]. Again, abscission arrest is dependent upon CHMP4C [106] and defects in NPC assembly produce aberrant activation of Aurora B [136], which may inhibit ESCRT-III assembly or function through CHMP4C. The mechanism by which impaired assembly of NPCs during mitotic exit results in subsequent impairment of ESCRT-III activity during cytokinesis remains unclear, however the involvement of ANCHR [129], ULK3 [108] and CDC-Like Kinases (CLKs) [138] in this pathway suggests that these defects are fed into the canonical abscission checkpoint. Whatever the mechanism, it appears that the abscission checkpoint is engaged to ensure cells enter G1 with functional NPCs.

In yeast, a related situation exists whereby malformed NPCs accumulate in the absence of ESCRT-III activity [139]. Budding yeast, where this phenomenon was originally observed, display a closed mitosis and divide their duplicated genome without needing to disassemble and reassemble their nuclear envelopes. It is possible that the need to continually remodel and insert new NPCs without having the luxury of *de-novo* insertion during open mitosis has forced the adoption of this surveillance strategy in these organisms. Again, the exact mechanism of surveillance remains to be established, with suggestions that ESCRT-III functions either to extract, or cause budding of these structures into the intermembrane space [140]. However, recent data suggesting that ESCRT-III functions in sealing off the nuclear envelope around the defective NPCs, aligns the biology of this process with that observed for nuclear envelope sealing in mammalian cells [141] and is discussed later.

It is important to note that there is unexplained variability in the outcome of disrupting the abscission checkpoint, even within similar experimental systems. Sustained Aurora B activity was originally observed to stabilise intercellular canals and prevent tetraploidisation induced by cleavage furrow regression [109], whereas rendering CHMP4C unresponsive to Aurora B phosphorylation allows abscission to proceed unchecked [106]. Aurora B’s role thus seems to be to stabilise the midbody and prevent it from being severed during abscission. Indeed, through phosphorylation of the microtubule plus-end binding protein End Binding Protein-3 (EB3) on S176, Aurora B can promote midbody stability and prevent premature midbody disassembly [142], thus as well as retarding assembly of the abscission machinery, engagement of the abscission checkpoint could act to stabilise the intercellular bridge. However, whilst depletion of other components of the abscission checkpoint such as ULK3 permits unchecked cytokinesis completion [108], depletion of ANCHR [129], or in some systems, CHMP4C [129], causes cleavage furrow regression and multinucleation. It is fair to say that we don’t fully understand the consequences of inactivation of the abscission checkpoint and these different outcomes need to be better understood. Whilst Aurora B phosphorylation of CHMP4C has been thought to play a major role in effecting an abscission checkpoint, the Mitotic Kinesin-Like Protein-1 (MKLP1), itself proposed as a tether between the cleavage furrow and the cortex [143], may also be an important regulator of this checkpoint. MKLP1 localises to chromatin bridges and is phosphorylated by Aurora B. One function of persistent Aurora B activity may thus be to retain anchors between the cleavage furrow and cortex to prevent regression and multinucleation [109]. In this context, it is interesting that MKLP1 forms part of the centralspindlin complex (MKLP1/MgcRacGAP), itself responsible for CEP55 and subsequently ESCRT-III recruitment to the midbody [46], [84], [86], and which tethers the plasma membrane to the intercellular bridge through interaction of MgcRacGAP’s C1 domain with membrane lipids [144]. Further, MKLP1 itself bound CHMP4C and its depletion interfered with detection of phosphorylated CHMP4C at the midbody, suggesting problems in recruitment of CHMP4C, or its phosphorylation in the absence of MKLP1, which may due in part to aberrant spindle organisation after MKLP1 depletion [130]. The related kinesin MKLP2 is necessary for relocalisation of the Chromosomal Passenger Complex components Aurora B and the Inner Centromere Protein (INCENP) from chromatin in anaphase to the central spindle and midbody [145], however, MKLP2 itself appears a target of Aurora B. Aurora B-dependent phosphorylation of MKLP2 in a newly identified membrane-binding region inhibited abscission and promoted furrow regression [146]. B56-PP2A, a key phosphatase in the regulation of mitotic exit, could counter Aurora B’s phosphorylation of MKLP2 to accelerate abscission [146]. These data suggest that the interplay between kinase and phosphatase activity, particularly within membrane anchoring regions of these essential cytokinetic motors, may contribute to the differential outcomes (furrow regression versus cytokinesis completion) observed when interfering with different components of this checkpoint.

## 11 ESCRT-dependent cytokinesis in animals

Whilst ESCRTs have a clear role in cytokinesis in mammalian cells and flies and are necessary for division in *Archaea*, they appear dispensable for cytokinesis in a number of organisms such as yeasts and *C. elegans*. In worms, high resolution tomographic approaches investigating the 1st mitotic division in the *C. elegans* embryo have demonstrated that membrane sealing occurs around the midbody after furrow ingression, leading to cytoplasmic separation [147], [148], a step shown to be independent of ESCRTs [148]. ESCRTs were instead observed to contribute to shedding of the midbody ring into the posterior cell [148]. This recent study instead implicated ESCRTs alongside actin and dynamin in a membrane remodelling process that also acted to remove membrane from the midzone during cytokinesis, rather than the actual process of abscission itself. Indeed, in worms depleted of Tsg101, abscission still occurred, although it was delayed [147]. These data suggest an abscission-independent role for ESCRTs in worm cytokinesis. As discussed above, in *D. melanogaster*, ALIX and ESCRT-III are necessary for cytokinetic abscission of germline stem cell bridges *in-vivo* [78], [125]. Moreover, in mammals, ESCRT-dependent abscission is specifically inhibited through the expression of Testes Expressed-14 (TEX14), a germ-cell specific protein that mimics the GPP motif within TSG101 and ALIX that are normally recruited by CEP55 [149]. In this manner, TEX14 expression ensures abscission is disrupted to enable the generation of stable intercellular bridges in male germ cells [149], which is necessary for proper spermatogenesis and organismal fertility [150], [151]. How (and whether) this stabilisation of intercellular bridges through TEX14-dependent competition for ESCRT-recruitment is integrated with stabilisation of these bridges through Aurora B engagement remains to be established. *TEX14* is found in a locus that contributes to the susceptibility for Testicular Germ Cell Tumours [152] and it will be important to determine whether control of ESCRT-dependent abscission affects the development of germ cell tumours. Moreover, already implicated in control of the cytokinesis checkpoint, cells lacking CHMP4C accumulate DNA damage and CHMP4C has been identified recently as a susceptibility locus for ovarian cancer [106], [153]. It will be important to determine whether CHMP4C acts as a tumour suppressor by regulating the abscission checkpoint and controlling ESCRT-III-dependent abscission. These indicate physiologically relevant roles for ESCRT-dependent cytokinesis and highlight the cellular and organismal dysfunction that can occur when its normal function is compromised.

## 12 ESCRT-III-dependent nuclear envelope sealing

Accepted roles for ESCRT-III as the cytokinetic abscission machinery have, in recent years, become established. However, early reports indicating the presence of CHMPs on mitotic chromatin [57] suggested that additional mitotic roles for ESCRT-III may exist. As explained above, the open mitosis performed by metazoans requires disassembly of the nuclear envelope at mitotic onset, and reformation in individual daughter cells during mitotic exit. Following chromatid separation, individual daughter nuclei are regenerated during anaphase and telophase. This requires disassembly of the mitotic spindle, decondensation of mitotic chromatin and envelopment of daughter nuclei by cellular membranes to form a bi-membraned nuclear envelope.

It was initially suggested that daughter nuclear membranes were formed *de-novo* from vesicles produced during nuclear envelope fragmentation at prophase. However, this theory was developed in *X. laevis* cell-free extracts, within which, unlike in intact cells, membranes begin in a fragmented state. It is now more widely accepted that the ER is the source of nuclear membranes, with contact sites between ER tubules and chromatin observed shortly after anaphase, followed by the coating of chromatin with ER membranes [13], [154], [155]. As cells leave mitosis, membranation of the chromatin is driven by interaction of nuclear envelope specific transmembrane proteins with chromatin and chromatin-associated factors which provide temporal control over the envelopment process, allowing the flattened cisternal ER sheets to envelope the chromatin. In in-vitro reconstitution assays, p47 and p97-dependent fusion events allows sheet expansion to accommodate the growing nuclei [156]. These sheets must negotiate spindle microtubules which traverse the nascent nuclear envelope, and must themselves be disassembled. Where these sheets meet in three dimensions, they leave a hole that is sealed in a topologically specialised process called annular fusion [18], [157], allowing generation of a sealed nuclear envelope and the re-establishement of nucleo-cytoplasmic compartmentalisation (Fig. 2A and B).

In recent years, a startling relocalisation of ESCRT-III to the reforming nuclear envelope has been observed for a brief period during mitotic exit [158], [159]. ESCRT-III localisation to this organelle occurs prior to reestablishment of the lamina, reincorporation of NPC components and reinitiation of nucleocytoplasmic transport, and appears to coincide with the completion of annular fusion. Endogenous ESCRT-III components localised transiently, and in a punctate manner, to nuclear membranes with high-resolution 3D-electron tomographic and super-resolution approaches revealing that these punctae represented assembly at sites of annular fusion [158], [159]. Satisfyingly, completion of annular fusion by resolving the stalk connecting inner and outer nuclear membranes bears a topologically equivalence to all ESCRT-III dependent membrane remodelling events. Here, ESCRT-III acts to separate the inner nuclear membrane (INM) and outer nuclear membrane (ONM), creating a sealed nuclear envelope [6], [7] (Fig. 2A–C). ESCRT-III assembly at sites of annular fusion lasted for only a few minutes, which, whilst brief, is consistent with the timescale of ESCRT-III assembly during viral budding [104], [160] and the terminal phases of cytokinesis [93]. Depletion of ESCRT-III components results in the persistence of unsealed holes in the nuclear envelope which led to a breakdown of nucleo-cytoplasmic compartmentalisation, evidenced by impaired sequestration of Nuclear Localisation Sequence (NLS)-targeted probes [158], [159]. This disruption in nuclear envelope integrity led to the presence of DNA damage foci at sites associated with unsealed holes in the nuclear envelope, indicating that ESCRT-III plays an important role in maintaining genome stability [159]. This function in closing holes in the nuclear envelope is particularly relevant during constrained migration [161], [162], [163], where interphase cells employ this machinery to repair ruptures to this organelle and maintain cellular viability [164].

Nuclear envelope reformation around newly segregated chromatin occurs in parallel with the disassembly of spindle microtubules which traverse the nuclear envelope. Mechanisms to remove these microtubules remained unclear until recently, but the role of ESCRT-III in sealing nuclear membranes provides a route through which this could be accomplished. As in cytokinesis, ESCRT-III subunits can recruit the microtubule severing AAA-ATPase, Spastin to disassemble these fibres. In the context of nuclear envelope reformation, Spastin recruitment is dependent upon its MIT-domain and on the presence of IST1 [159]. The significance of this cross-talk between ESCRT-mediated nuclear membrane sealing and Spastin-mediated spindle disassembly is apparent as a delay in microtubule recycling is seen in ESCRT-III depleted cells, with microtubules remaining attached to the chromatin disc at sites associated with ESCRT-III foci [159].

Due to the numerous membrane remodelling roles of ESCRT-III in the cell, the machinery must be recruited to its site of action by location-specific adaptor proteins. As described, in the case of cytokinesis, ESCRT-III is recruited by the CEP55 midbody protein and during viral budding and multivesicular body biogenesis it is recruited by Gag and ESCRT-0 respectively. In the context of nuclear envelope reformation, the Stenmark laboratory identified that the poorly studied ESCRT-III protein, CHMP7, was necessary for ESCRT-III assembly at the nuclear envelope, pointing to a specific role for this protein in this specialised process [159]. In addition to CHMP7, the p97-associated factor Ubiquitin Fusion Degradation-1-Like (UFD1L) is able to contribute to incorporation of late-acting ESCRT-III components to these sites of annular fusion [158]. In-vitro reconstitution assays have implicated Ubiquitin Fusion Degradation-1 (UFD1) and its partner, Nuclear Protein Loclaisation-4 (NPL4) in annular fusion [156], but whilst UFD1L can interact with CHMP2A, the mechanism by which it stimulates CHMP2A assembly at sites of annular fusion remains unclear.

CHMP7 is somewhat of a hybrid protein, with a C-terminal CHMP-like domain that resembles other ESCRT-III subunits and an N-terminal tandem winged-helix (WH) domain, which resembles well the tandem-WH domain containing, ESCRT-II subunits [141], [165], [166]. In the context of endosomal sorting, ESCRT-II acts to recruit ESCRT-III to endosomal membranes [167] through interactions with 3-phosphoionositides [32], [36], and recent data suggest that the ESCRT-II-like domain of CHMP7 acts similarly to recruit ESCRT-III to cellular membranes − in this case, the ER and nuclear envelope.

In mammalian cells, endogenous CHMP7 localises to the ER [166], which, during mitotic coalescence with the nuclear envelope, allows a platform to recruit ESCRT-III to the reforming nuclear envelope. CHMP7′s N-terminus contains an evolutionarily conserved stretch of hydrophobic amino acids which function as a membrane anchor to assemble ESCRT-III at this organelle. Disruption of this membrane anchor renders CHMP7 cytoplasmic and unable to initiate ESCRT-III assembly, demonstrating the essential nature of these protein-lipid interactions for closing holes in this organelle. These data also indicate that the CHMP-like domain is insufficient for assembling ESCRT-III at sites of annular fusion [166]. What, then, may specify recruitment of CHMP7 to sites of annular fusion in the reforming nuclear envelope? Recent data from the Frost, Sundquist and Ullman laboratories has implicated the INM protein LEM2 in initiating recruitment of ESCRT-III to the nuclear envelope through binding directly to CHMP7 [168]. Assembly of CHMP7, CHMP2A and IST1 at the reforming nuclear envelope was found to be dependent upon LEM2 [168]. These data again highlight how ESCRT-III acts as a transplantable membrane remodelling machinery that in this case employs co-incidence detection [169] of membrane cues and INM proteins such as LEM2 to specify the site of recruitment. Indeed, the co-incidence detection of mitotic ER and chromatin-bound INM proteins may impose a spatial constraint upon ESCRT-III assembly to restrict it to sites of continuity between INM and ONM (Fig. 2C).

Whilst yeast typically undergo a closed mitosis, they too express a CHMP7 homologue: Chm7 in *S. cerevisiae* and Cmp7 in *S. pombe*, with data suggesting an ER function for this protein [165]. Importantly, whilst these yeast undergo a closed mitosis, they must still divide their nuclei and nuclear envelopes in two, which will necessitate a resealing activity – whether yeast ESCRTs function in this process remains to be established. Fission yeast lacking Vps4 exhibit fenestrated, morphologically abnormal and poorly sealed nuclear envelopes, which, given their closed mitosis, suggests a more global role in nuclear envelope homeostasis for these proteins. Intriguingly, these phenotypes could be suppressed by spontaneous loss of function mutations in either Cmp7 or Lem2 [168]. These data indicate that if ESCRT-III can’t be recruited to this organelle, the deleterious consequences of ESCRT-inactivation on nuclear envelope phenotypes are not exposed and suggest that alternate mechanisms for sealing the nuclear envelope may operate in this case.

## 13 ESCRT-III-dependent NPC surveillance at the nuclear envelope

**Fig. 5:**
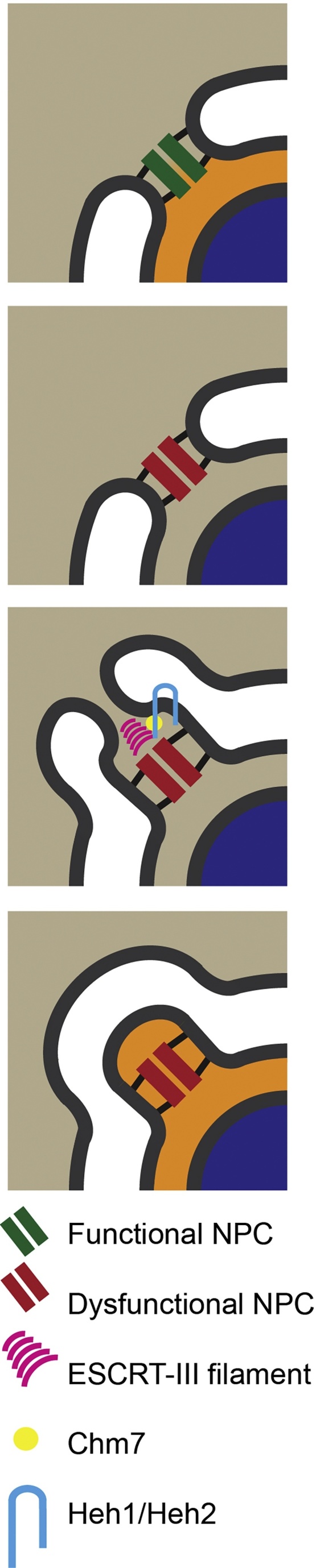
Potential role for Chm7 and Heh2 in surveillance of damaged NPCs. Mis-assembled NPCs are cleared in an ESCRT-III and proteasome dependent manner. In one model of misassembly, NPCs lacking Nup116 are encapsulated by new membrane to seal them off and prevent them participating in nucleocytoplasmic transport. The N-terminal domain of the INM LEM-domain protein Heh2 interacts with both Snf7 and the CHMP-like C-terminus of Chm7 and may play a role in this, from an ESCRT-III perspective, topologically satisfying closure. Hypothesis and figure suggested by Lusk and colleagues and drawn and adapted from [141], [170].

As described earlier, ESCRT-III proteins also act at the nuclear envelope of *S. cerevisiae*, albeit during interphase. Here, they act to survey for mal-assembled, or poorly functioning NPCs, whose existence would lead to a breakdown in nucleocytoplasmic compartmentalisation. These NPCs are cleared in a manner requiring ubiquitin and the proteasome, and in strains lacking ESCRT-III components, these defective NPCs aggregate into a single focus, deemed the Storage of Improperly Assembled NPCs (SINC) compartment. Once more, the LEM-domain-containing INM proteins are implicated in this control; Heh2 can bind to early NPC assembly products, and acts to recruit Snf7/CHMP4 and Vps4, indicating that ESCRT-III function acts somehow to remove these defective NPCs [139]. Recently, Chm7 has been shown to be directly involved in clearing these defective NPCs, being recruited to the anomalous SINC compartment through the LEM-domain containing proteins Heh1 and Heh2 [141]. Lusk and colleagues used yeast bearing a temperature-sensitive mutation in Nup116–in this strain, the defective NPCs are thought to become sealed off by new nuclear membrane [170], a process that bears topological equivalence to ESCRT-III mediated membrane sealing. Chm7 maintained the viability of strains bearing compromised NPCs, suggesting that Chm7 and ESCRT-III may act to seal fresh nuclear membrane over the mis-functioning NPC, thus preventing it participating in barrier functions [141] (Fig. 5). The defective NPC would now likely be found in a new INM, where established proteostasis mechanisms such as the Asi complex or other INM-relevant ER-associated degradation mechanisms [171], [172] could act to recycle these components. Deleting Chm7 resulted in compartmentalisation breakdown in strains bearing defective NPCs, indicating how Chm7 contributes to cellular viability. These data have the potential to rationalise these different ESCRT-III functions at the nuclear envelope, and highlight again ESCRT-III’s role as a membrane remodelling machinery.

It is unclear whether a similar surveillance role exists in mammalian cells, given that they have ample opportunity to remodel their NPCs during each mitosis. However, as described above, telling data indicating that NPC mis-assembly can signal through Aurora B to the ESCRT-III mediated abscission checkpoint, to impose a temporal delay upon cytokinesis, suggests that such a quality control mechanism may exist [136], [137]. Entering the next G1 with improper control over nucleocytoplasmic compartmentalisation would likely be detrimental for cellular viability, and whether a NPC-associated quality control system exists to suppress cytokinetic ESCRT-III assembly remains an exciting area of future study.

A number of important questions regarding ESCRT function on the nuclear envelope remain. We have little understanding of the lipid environment necessary for CHMP7 to assemble ESCRT-III and, whilst interaction with LEM-domain containing proteins is important, we don’t fully understand how ESCRT-III assembly is restricted to sites of annular fusion in the nuclear envelope subdomain. Interaction with spindle microtubules is likely important, but the finding that ESCRT-III operates to repair interphase nuclear envelope ruptures, which likely occur in the absence of traversing microtubules, suggests that additional factors may be required. Our understanding of how membrane remodelling is coordinated with insertion of NPCs [173] remains unclear and whether NPC surveillance occurs in mammalian cells remains an open question. Topologically equivalent membrane remodelling is necessary for a variety of viruses such as herpesviruses and large Ribonuclear Protein granules to traverse the nuclear envelope [174]. It is tempting to speculate that ESCRT-III is involved in this transport, particularly in budding across the INM into the intermembrane space, as this step appears topologically suited to ESCRT-III function on this organelle. The adaptor protein ALIX has been implicated in nuclear envelope transit for the herpesvirus Epstein Barr Virus (EBV), being able to interact with the viral protein BFRF1 [175]. Ubiquitin and the ESCRT-associated ubiquitin ligase, ITCH, play roles too in the maturation of EBV at the nuclear envelope [176]. ESCRTs had previously been implicated in the secondary envelopment of herpesvirus particles [177], however, it remains possible that they too contribute to nuclear envelope passage too. The coming years look likely to illuminate much exciting ESCRT biology upon this organelle.

## 14 Unexplored roles for the ESCRT machinery in mitosis

Mitotic roles for ESCRTs have largely stemmed from their ability to remodel membranes, however, intriguing findings suggest that more expansive mitotic roles may be played by these proteins. Prior to its relocalisation to the midbody, CEP55, the key recruiter of ESCRT-III, localises to centrosomes [86], [87]. VPS4 was similarly reported to localise to mitotic centrosomes, suggesting there may well be unexplored functions for ESCRTs on this membrane-less organelle [178]. As well as abscission defects, ESCRT-depletion resulted in disruption of the centrosome cycle, with depletion of most ESCRT-III members causing fragmentation of centrosomes and the pericentriolar material. Loss of CHMP5 or CHMP2A, however, was reported to result in a prevalence of monopolar spindles, suggesting that ESCRTs may regulate both accumulation and dispersal of centriolar material. In many cases, defects in mitotic chromosome alignment were produced which masked subsequent abscission defects and/or produced morphologically abnormal nuclei [178]. It is not currently clear what function ESCRTs are playing on centrosomes and how they regulate the integrity of this organelle. Indeed, the nuclear morphology defects observed upon ESCRT suppression, and currently ascribed to segregation errors, may instead relate to impaired nuclear envelope biology. However, in *S. pombe* lacking Vps4, karyokinesis defects were observed and the duplicated nuclear envelope-embedded spindle pole bodies failed to separate [168], suggesting the involvement of ESCRTs in genome segregation in this system too. These cells displayed extensive karmallae (stacks of ER and nuclear envelope membrane that surround the nucleus) and excessive tubular extensions and nuclear envelope fenestrations, indicating a breakdown of proper nuclear envelope remodelling in the absence of Vps4 and suggesting that control of the nuclear envelope and spindle pole biology is intimately linked [168].

## 15 Concluding remarks

The prevalence of ESCRT functions in recent years has taken many by surprise; it truly feels as though ‘ESCRTs are everywhere’ [9]. However, the topological similarities between all ESCRT-III regulated events point to the evolutionary conservation of these proteins as a specific membrane remodelling machinery that is deployed to various cellular membranes to effect completion of a biological process. The degree of involvement of ESCRT-III in mitotic membrane remodelling events is striking and its evolutionary conservation as far back as *Archaea* suggests that mitotic functions were the ancestral role of this machinery that has been co-opted throughout evolution to effect completion of a variety of cell biological processes. These processes all require the resolution of a cytoplasm filled stalk connecting two membranes, with ESCRT-III acting to sever of this stalk. Telling cartoons hypothesise the narrowing of an ESCRT-III spiral filament bringing the walls of these stalks closer together until wall-fusion becomes inevitable, but it would be prudent to remember that we have very little idea of how the membrane fission reaction actually occurs. We do know that ESCRT-III acts to separate membranes previously tethered by stalks (intraluminal vesicle and endosomal limiting membranes during ILV biogenesis; viral and plasma membranes during viral release; daughter cell plasma membranes during cytokinesis and the INM and ONM during nuclear envelope repair and reformation). It is hoped that biophysical lines of enquiry will soon shed light on the mechanics by which these membrane fission reactions are performed.

Mitotic biology relies upon proper completion of precisely temporally coordinated events, and the surprising involvement of ESCRTs in a wealth of mitotic processes will hopefully provide routes by which regulation and activation of ESCRT activity can be explored. Documented roles for ESCRTs in nuclear envelope biology and cytokinesis have been established, although whether these processes are linked by co-regulatory mechanisms remain unclear. For example, it will be important to discover if deregulated ESCRT biology at the nuclear envelope engages the abscission checkpoint, or, for example, whether there is coordination between nuclear envelope sealing on lagging chromosomes and engagement of this checkpoint. For nuclear envelope-relevant ESCRT-function, it will also be important to examine how membrane sealing is coordinated with functionalisation of this organelle through re-establishment of NPC function and regeneration of INM and ONM identity, and to understand further the evolutionary conservation of this function. Regards cytokinesis, a scaling problem between the staging of ESCRT-III at the relatively large midbody ring and its eventual transition to the abscission site needs to be understood, as does the relatively unexplored process of midbody maturation. Moreover, the apparent absence of involvement of ESCRTs in abscission in yeast or *C. elegans* leads us to question how these species complete cytokinesis. These are exciting times for ESCRT-biology and it is hoped that our deeper understanding of roles for this machinery in cell division will illuminate the workings of fundamental cellular processes that are essential for life, growth and development.
